# Microglial p38α MAPK is a key regulator of proinflammatory cytokine up-regulation induced by toll-like receptor (TLR) ligands or beta-amyloid (Aβ)

**DOI:** 10.1186/1742-2094-8-79

**Published:** 2011-07-06

**Authors:** Adam D Bachstetter, Bin Xing, Lucia de Almeida, Edgardo R Dimayuga, D Martin Watterson, Linda J Van Eldik

**Affiliations:** 1 Sanders-Brown Center on Aging, University of Kentucky, Lexington, KY, USA; 2 Dept of Celland Molecular Biology, Northwestern University Feinberg School of Medicine, Chicago, IL, USA; 3 Dept of Molecular Pharmacology and Biological Chemistry, Northwestern University Feinberg School of Medicine, Chicago, IL, USA; 4 Dept Anatomy and Neurobiology, University of Kentucky, Lexington, KY, USA

**Keywords:** Microglia, cytokines, toll-like receptors, knockout mice, p38alpha mitogen-activated protein kinase, amyloid beta-peptides, drug discovery

## Abstract

**Background:**

Overproduction of proinflammatory cytokines from activated microglia has been implicated as an important contributor to pathophysiology progression in both acute and chronic neurodegenerative diseases. Therefore, it is critical to elucidate intracellular signaling pathways that are significant contributors to cytokine overproduction in microglia exposed to specific stressors, especially pathways amenable to drug interventions. The serine/threonine protein kinase p38α MAPK is a key enzyme in the parallel and convergent intracellular signaling pathways involved in stressor-induced production of IL-1β and TNFα in peripheral tissues, and is a drug development target for peripheral inflammatory diseases. However, much less is known about the quantitative importance of microglial p38α MAPK in stressor-induced cytokine overproduction, or the potential of microglial p38α MAPK to be a druggable target for CNS disorders. Therefore, we examined the contribution of microglial p38αMAPK to cytokine up-regulation, with a focus on the potential to suppress the cytokine increase by inhibition of the kinase with pharmacological or genetic approaches.

**Methods:**

The microglial cytokine response to TLR ligands 2/3/4/7/8/9 or to Aβ_1-42 _was tested in the presence of a CNS-penetrant p38α MAPK inhibitor, MW01-2-069A-SRM. Primary microglia from mice genetically deficient in p38α MAPK were used to further establish a linkage between microglia p38α MAPK and cytokine overproduction. The *in vivo *significance was determined by p38α MAPK inhibitor treatment in a LPS-induced model of acute neuroinflammation.

**Results:**

Increased IL-1β and TNFα production by the BV-2 microglial cell line and by primary microglia cultures was inhibited in a concentration-dependent manner by the p38α MAPK-targeted inhibitor. Cellular target engagement was demonstrated by the accompanying decrease in the phosphorylation state of two p38α MAPK protein substrates, MK2 and MSK1. Consistent with the pharmacological findings, microglia from p38α-deficient mice showed a diminished cytokine response to LPS. Further, oral administration of the inhibitor blocked the increase of IL-1β in the cerebral cortex of mice stressed by intraperitoneal injection of LPS.

**Conclusion:**

The p38α MAPK pathway is an important contributor to the increased microglial production of proinflammatory cytokines induced by diverse stressors. The results also indicate the feasibility of targeting p38α MAPK to modulate CNS proinflammatory cytokine overproduction.

## Background

Microglia, the resident macrophages of the central nervous system (CNS), monitor their environment through a constant movement of their processes, and respond to local stressors and immune disturbances [1,2]. Microglia express a complement of pattern recognition receptors (PRR) that can respond to pattern associated molecular patterns (PAMPs) and damage associated molecular patterns (DAMPs), such as Lipopolysaccharides (LPS) and β-amyloid (Aβ). A major class of PRRs includes the Toll-like receptors (TLRs) that play a pivotal role in host defense by regulating innate immunity and linking with adaptive immune responses (for reviews, see: [3,4]). Activation of TLRs on microglia leads to the production of inflammatory mediators, such as IL-1β, IL-6, TNFα, and nitric oxide. TLR engagement and signaling in the CNS provide an important defense mechanism by which microglia respond to external pathogens or host-derived ligands. Microglia can also be activated by inflammatory mediators (e.g. cytokines and chemokines) from autocrine, paracrine, and endocrine sources (for detailed reviews on microglia, see: [5,6]). The local environment, and possibly intrinsic changes to the microglia determine how the cells will respond to the activating signals [7,8]. Like peripheral immune cells, microglia can adopt a number of activated phenotypes, and the functional outcome depends on a complex balance between beneficial protective responses and detrimental harmful responses [9]. Tight regulation of microglial activation pathways is essential for appropriate responses to stressor stimuli and maintenance of CNS homeostasis, because uncontrolled or dysregulated inflammatory responses can lead to propagation of detrimental and neurotoxic responses.

A relevant example is the control of microglia proinflammatory cytokine production in response to various ligands. Proinflammatory cytokines have many important physiological functions in the CNS, from protection against pathogens to acting as neuromodulators affecting cognition [10]. However, clinical studies and preclinical animal models have implicated dysregulation and overproduction of proinflammatory cytokines from activated microglia in the CNS as a contributor to pathophysiology progression in both chronic neurodegenerative disorders such as Alzheimer's disease (AD), Parkinson's disease, and multiple sclerosis, as well as acute neurodegenerative conditions such as traumatic brain injury and stroke [11-13]. Taken in its entirety, the evidence is consistent with the hypothesis that proinflammatory cytokine overproduction is a comparatively early event in the progression of pathophysiology that is causally linked to synaptic dysfunction, behavior deficits and, in the more extreme case, neuronal death. This raises the possibility that up-regulation of proinflammatory cytokine production could be targeted in new therapeutic development strategies with potential for disease modification in multiple diseases and clinical presentations.

One approach to targeting CNS cytokine dysregulation is to modulate the intracellular signal transduction cascades that regulate the production of proinflammatory cytokines. This requires that we explore which specific signal transduction pathways are involved in cytokine overproduction in microglia exposed to different stressors, and which of these pathways are amenable to intervention. A major signaling pathway that contributes quantitatively to up-regulated cytokine production in peripheral inflammation is the p38 mitogen activated protein kinase (MAPK) pathway, especially the key regulatory enzyme p38α MAPK [14,15]. The p38α MAPK is amenable to therapeutic intervention in peripheral inflammatory diseases, and treatment with p38α MAPK-targeted inhibitors can suppress cytokine levels back towards basal. There is increasing evidence that the p38 MAPK signaling cascade also contributes to CNS cytokine overproduction and neurodegenerative sequelae, and that it may be a good therapeutic target for CNS disorders characterized by increased proinflammatory cytokine production as a contributor to neurologic dysfunction or susceptibility to injury [11,16]. However, studies are needed to determine a direct linkage between the p38α MAPK-mediated pathway in microglia and proinflammatory cytokine production in response to different stressors. In addition, whether the pathway in microglia is druggable; i.e., responsive to intervention with CNS-penetrant kinase inhibitors, needs to be determined.

The field has been limited in its ability to pursue these questions because of the lack of selective p38α MAPK inhibitors with sufficient brain penetrance and metabolic stability for use in CNS disorders [17]. We recently developed a selective, orally bioavailable, CNS-penetrant, small molecule p38α inhibitor, MW01-2-069A-SRM (069A) [18]. Compound 069A attenuates hippocampal proinflammatory cytokine overproduction and leads to improved neurologic outcomes in an AD-relevant mouse model when administered orally at a low dose and in a clinically relevant time window of disease progression; specifically, 069A suppressed the Aβ_1-42_-induced hippocampal IL-1β and TNFα up-regulation back towards basal and attenuated the resultant synaptic dysfunction and behavioral deficits [18]. These data provided an initial causative link between p38α MAPK and CNS disease-related endpoints. Moreover, pharmacological downregulation of IL-1β production may have important protective consequences, as IL-1β can induce the expression and activation of p38 MAPK in neurons, promoting tau phosphorylation and loss of the synaptic protein synaptophysin [19,20]. The availability of this p38α MAPK inhibitor, as well as a novel mouse model where p38α MAPK is genetically deleted in microglia, provide the opportunity to test whether p38α MAPK is a major regulator of microglial proinflammatory cytokine overproduction in response to specific stressors, and whether this signaling pathway is amenable to intervention. We report here that p38α MAPK is a key contributor to microglia proinflammatory cytokine production in response to a variety of stressor stimuli, including Aβ_1-42 _and different TLR ligands. In addition, inhibition of p38α MAPK by either pharmacological or genetic approaches leads to a reduction in microglia cytokine production. The results indicate the feasibility of targeting p38α MAPK in attempts to modulate proinflammatory cytokine overproduction, a potential contributor to CNS disease progression and susceptibility.

## Methods

### Reagents

#### Toll-like receptor (TLR) ligands

Lipopolysaccharides (LPS) from *Salmonella enterica serotype typhimurium *(Sigma-Aldrich, St. Louis, MO: Cat. no. L6143-1MG; EU/MG of LPS is 600,000) was prepared in sterile 0.9% sodium chloride. The following TLR ligands were prepared according to the manufacturer instructions (InvivoGen, San Diego, CA): the TLR2 ligand, lipoteichoic acid (LTA) from *Staphylococcus aureus *(10 μg/ml), (cat. no. tlrl-pslta); TLR-3 ligand, polyinosine-polycytidylic acid (poly(I:C)) low molecular weight (1mg/ml)(0.2-1kb; cat. no. tlrl-picw) and a high molecular weight (50 μg/ml) (1.5-8kb; cat. no. tlrl-pic); TLR7/8 ligand, CL097, (500ng/ml)(cat. no. tlrl-c97); and TLR 9 ligand, type B CpG oligonucleotide, ODN1668 (500ng/ml)(cat. no. tlrl-modnb).

#### Oligomeric Aβ_1-42_

Oligomeric Aβ_1-42 _was prepared from recombinant Aβ_1-42 _peptide (rPeptide, Athens, GA: cat. no. A-1002-2) as previously described [21,22]. Briefly, the Aβ peptide was monomerized in 1mM hexafluoroisopropanal (HFIP) (Sigma-Aldrich, St. Louis, MO), and the HFIP was evaporated using a SpeedVac. The dried peptide film was stored in a desiccator at -20°C until use. Oligomeric Aβ was prepared by resuspending the peptide in 5 mM dimethylsulfoxide (DMSO) (Sigma-Aldrich, St. Louis, MO; cat. no. D2650). The peptide was then diluted to 100 μM in phenol red-free F12 media (Promocell, Heidelberg, Germany; cat. no. C-72119). The Aβ solution was incubated for 24 hrs on ice at 4°C to allow oligomer formation. Oligomeric Aβ was used at a final concentration of 5 μM for stimulation of rat microglia cultures. Vehicle control used the same volumes of DMSO and F12 media as was used to generate the Aβ peptide preparations and followed all steps as the peptide.

#### Small molecule p38α MAPK inhibitor synthesis and compound administration

**Figure 1:**
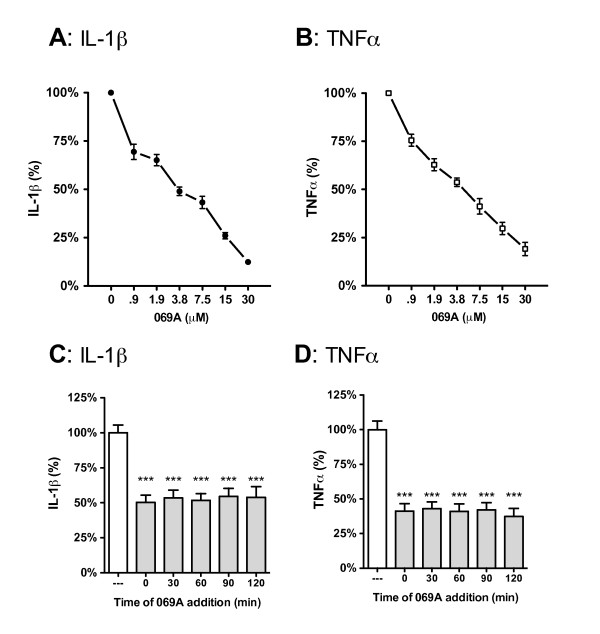
**The small molecule p38α MAPK inhibitor, 069A, suppresses the LPS-induced increase in cytokine production by the BV-2 microglia cell line**. BV-2 cells were stimulated with 100ng/ml of LPS in the absence or presence of increasing concentrations of 069A (0.9 - 30 μM). 069A inhibited LPS-induced IL-1β **(A) **and TNFα **(B) **production in a concentration-dependent manner, with an IC_50 _of 3.7 μM and 4.5 μM, respectively. Compound 069A (4 μM) was equally as effective in blocking the IL-1β **(C) **and TNFα **(D) **cytokine response to LPS when the compound was given at the same time as LPS (time 0) or when given at different times after LPS exposure. White bars show the response of the LPS stimulated cells in the absence of 069A. Gray bars show the response to LPS in the presence of 069A. Data are expressed as a percent of the maximal activity, where activity in the presence of LPS alone is taken as 100%. Asterisk denotes significance (*** = p < 0.001) in response to LPS in the absence (white bars) or presence (gray bars) of 069A.

The p38α MAPK inhibitor, 3-phenyl-4-(pyridin-4-yl)-6-(4-pyrimidin-2-yl)piperazin-1-yl)pyridazine (MW01-2-069A-SRM) (hereafter designated 069A) was synthesized according to the production scheme previously described [18]. Stock solutions of 069A were made in sterile 0.9% sodium chloride that was free of preservatives (Hospira, Inc., Lake Forest, IL: cat. no. NDC 0409-4888-10). Solutions for cell treatments were prepared by dilution of the stock solutions into serum-free media immediately before adding to the cells. Compound was added to cell cultures just before addition of the stressor stimulus, except for the experiment in Figure 1C, D that tested different times of compound addition. For *in vivo *experiments, mice received 069A (5 mg/kg) or the saline vehicle by oral gavage (po) in a volume of 200 μL one hr prior to the LPS injection.

### Cel Culture

Mixed glial cultures were prepared from the cerebral cortex of 1-2 day old neonatal C57Bl/6 mice or Sprague-Dawley rats as previously described [23]. The cells were maintained for 2-6 weeks in α-minimum essential medium (α-MEM) supplemented with 10% fetal bovine serum (FBS) (US Characterized FBS; Hyclone; Cat no. SH3007103N), 100 IU/ml penicillin, 100 μg/ml streptomycin (Mediatech Cat no. 30-002-CI) and 2mM L-Glutamine (Mediatech Cat no. 25-005-CI). Microglia were isolated from the mixed glial cultures by the shake-off procedure [24]. Briefly, the flasks were shaken for 2 hr at 250 rpm on an orbital shaker at 37°C, the media containing loosely adherent cells were harvested into 48-well plates at 1 × 10^5 ^or in a 12-well plate at 3 × 10^5^. After 30 min, nonadherent cells were removed and fresh serum-free medium supplemented with 5ng/ml M-CSF (R&D systems; Cat no. 416-ML) was added. The cells were then cultured for 24 hrs before treatment.

The murine microglial BV-2 cell line [25] was cultured in DMEM/F12 (Mediatech; Cat no. #15-090-CV) supplemented with 10% FBS, 100 IU/ml penicillin, 100 μg/ml streptomycin (Mediatech Cat no. 30-002-CI) and 2mM L-Glutamine (Mediatech Cat no. 25-005-CI). For experiments, serum-containing medium was removed and cells were treated with stimulus or vehicle control in serum-free medium. Cells were harvested after 1 hr of stimulation for Western blot analysis, and after 18 hrs of stimulation for cytokine measurements.

### Animals

All experiments were conducted in accordance with the principles of animal care and experimentation in the Guide For the Care and Use of Laboratory Animals. The Institutional Animal Care and Use Committees of the University of Kentucky and Northwestern University approved the use of animals in this study. Neonatal C57Bl/6 mice or Sprague-Dawley rats were used to make primary glial cultures. Male and female, 2-month-old, C57Bl/6 mice (Harlan) were used for the *in vivo *LPS experiment. The p38α conditional knockout mice were generated as previously described [26]. Briefly, the first exon of the p38α gene was flanked by two loxP sites, the floxed allele was bred to homozygosity, and then crossed with mice that contain Cre driven by the lysozyme promoter. The mice (all p38α^fl/fl ^and backcrossed to C57Bl/6) are maintained and bred as follows: Female mice that are p38^fl/fl ^wildtype and not carrying the Cre allele are bred with male mice that are Lys-Cre hemizygotes. This generates litters where ~50% mice are microglia p38α knockout and ~50% are p38α^fl/fl ^littermates (used as wild-type controls). Genotyping was performed by Transnetyx, Inc (Cordova, TN) to confirm mouse genotypes.

### Western blotting

BV-2 cells or primary microglia were plated in 12 well plates at a density of 1 × 10^5 ^cells/well or 3 × 10^5 ^cells/well, respectively. Western blotting analysis was performed as previously described [27]. Cell lysates were prepared in sodium dodecyl sulfate (SDS)-containing sample buffer and equal volumes of cell lysates were separated by 10% SDS-polyacrylamide gel electrophoresis (SDS-PAGE). After transfer to Immobilon-P membranes (Millipore) and blocking, blots were probed with the following primary antibodies from Cell Signaling Technology (Beverly, MA): rabbit anti-pMSK1 (cat. no. 9595 (1:1000)); rabbit anti-pMK2 (cat. no. 3041 (1:1000)); rabbit anti-pCREB (cat. no. 9191 (1:1000)); and rabbit anti-pATF2 (cat no. 9221 (1:1000)): mouse anti-pp38α/β (cat no. 9216 (1:1000)); rabbit anti-p38α/β (cat no. 9212 (1:1000)); rabbit anti-p38α (cat no. 9218 (1:1000)); mouse anti-β-Actin (Cat no. 3700 (1:10000)).

### In vitro cytokine measurements

**Figure 2:**
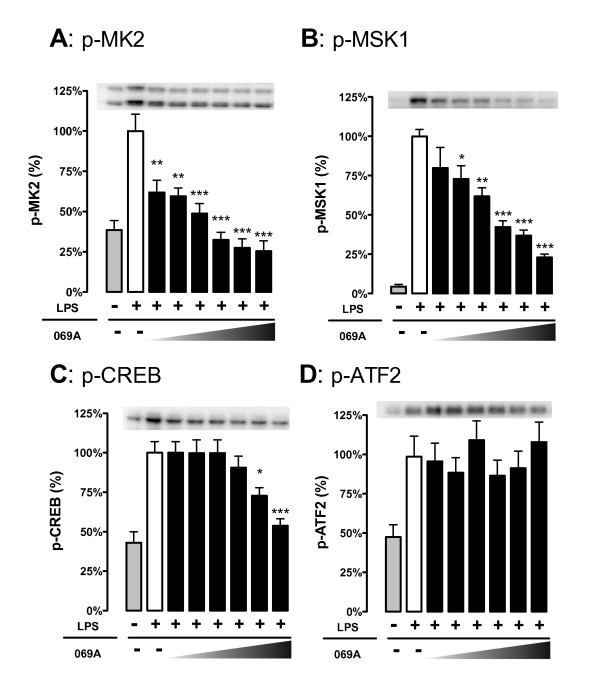
**The p38α inhibitor, 069A, selectively inhibits phosphorylation of the p38α substrates MK2 and MSK1 in LPS-stimulated BV-2 microglia cells**. BV-2 cells were stimulated with 100ng/ml of LPS in the absence or presence of increasing concentrations of 069A (0.9 - 30 μM). Levels of **(A) **p-MK2 and **(B) **p-MSK1 were suppressed by the p38α inhibitor in a concentration-dependent manner. Treatment of cells with 069A had an effect on p-CREB **(C) **only at the two highest compound concentrations, and had no effect on p-ATF2 levels **(D)**. Gray bars show the response of control, unstimulated cells; white bars show the response of LPS-stimulated cells in the absence of 069A; black bars show the response of LPS-stimulated cells in the presence of 069A. For each endpoint, data are expressed as a percent of the maximal activity, where activity in the presence of LPS alone is taken as 100%. Asterisk denotes significance (* = p < 0.05, ** = p < 0.01, or *** = p < 0.001) in endpoint for LPS-stimulated cells (white bars) to LPS-stimulated cells in presence of 069A (black bars). Data represent five independent experiments.

**Figure 3:**
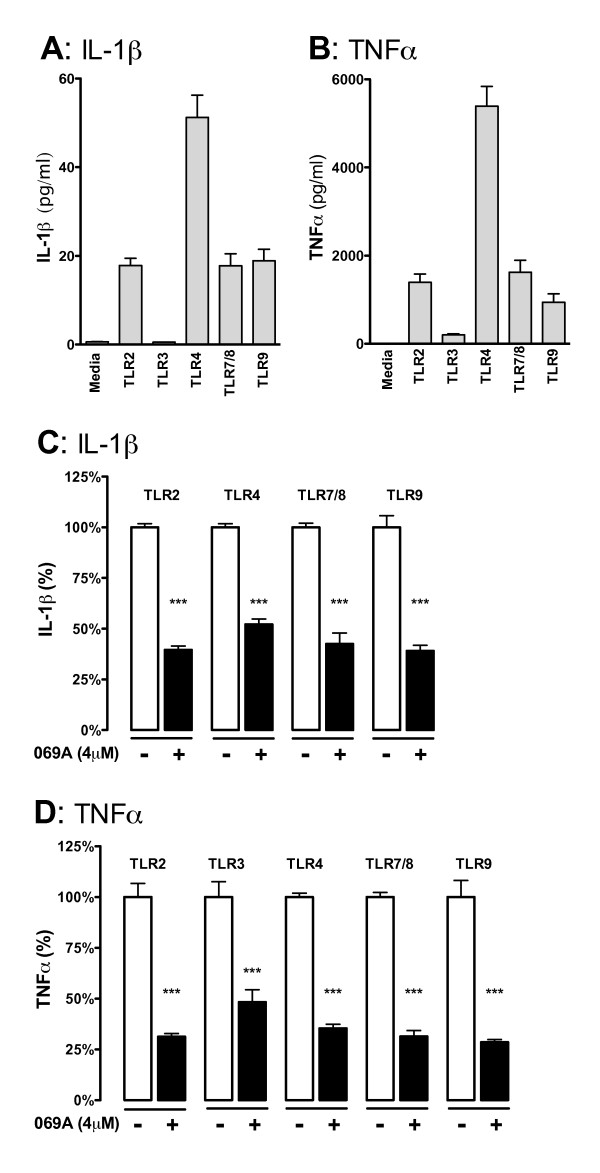
**The p38α MAPK inhibitor 069A attenuates the IL-1β and TNFα increase in response to diverse TLR ligands**. The IL-1β **(A) **and TNFα **(B) **response from BV-2 cells was measured after 18 hrs of stimulation with either: (TLR2) 10 μg/ml LTA; (TLR3) 50 μg/ml poly(I:C); (TLR4) 100ng/ml LPS; (TLR7/8) 500ng/ml CL097; or (TLR9) 500ng/ml ODN1668. Treatment of cells with 4 μM of the p38α inhibitor, 069A, led to a significant reduction in the levels of IL-1β **(C) **and TNFα **(D) **in response to each of the different TLR ligands. The white bar represents the BV-2 cells stimulated with the ligand but without 069A (normalized to 100%). The black bar represents the BV-2 cells treated with TLR ligand + 069A. Asterisk denotes significance (* = p < 0.05, ** = p < 0.01, or *** = p < 0.001) for ligand-stimulated without 069A (white bar) compared to stimulated with 069A (black bar). Data represent two independent experiments.

**Figure 4:**
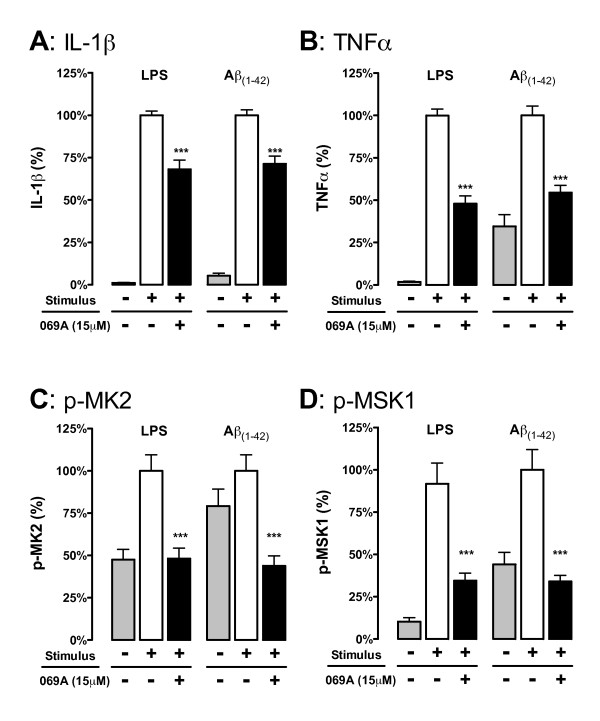
**The p38α MAPK pathway is engaged in primary microglia and contributes to cytokine upregulation**. Rat primary microglia were treated with either diluent (gray bars), stressor (either 1ng/ml LPS or 5 μM oligomeric Aβ_1-42_) in the absence of 069A (white bars), or stressor in the presence of 15 μM 069A (black bars). The LPS-induced increases in IL-1β **(A) **and TNFα **(B) **levels by rat primary microglia were significantly inhibited by the p38α inhibitor 069A. Similar results were obtained with a non-TLR ligand, Aβ_1-42_. LPS or Aβ also caused an increase in the phosphorylated (active) form of two p38α substrates, p-MK2 **(C) **and p-MSK1 **(D)**, and phosphorylation of these substrates was blocked by the addition of 069A. Asterisk denotes significance (* = p < 0.05, ** = p < 0.01, or *** = p < 0.001) for stressor-stimulated microglia in the absence of 069A compared to stimulated microglia in the presence of 069A. Values with stimulus alone (white bars) were normalized to 100%. Data represent four independent experiments.

After testing a number of different cell densities, a BV-2 cell density of 2 × 10^4 ^in a 48 well plate incubated for 24 hrs prior to addition of stimulus produced the most consistent cytokine response with the lowest intra- and inter-assay variability (data not shown). Primary microglia were used in 48-well plates at 1 × 10^5 ^cells. Levels of TNFα were measured in the conditioned media by ELISA using kits from Meso Scale Discovery (MSD; Gaithersburg, Maryland)(Figure 1&2) or R&D Systems (Minneapolis, MN) (Figure 3&4). For IL-1β, cell lysates were prepared in lysis buffer containing 20mM Tris, pH8; 2mM EDTA; 0.5% Triton X-100. Levels of IL-1β were measured in the conditioned media by ELISA using kits from MSD (Figure 1&2) or R&D Systems (Figure 3&4).

### Tissue collection and cytokine measurements

At 90 min and 6 hrs after LPS administration, mice were injected with an overdose of sodium pentobarbital (Pentasol powder; Vibrac Animal Health, Ft Worth, TX: cat. no. NDC-051311-103-25). Blood was collected for serum cytokine measurements. The mice were then decapitated. The brain was dissected on ice and snap-frozen in liquid nitrogen. The tissue was stored at -80°C until time of use. Brain cortex was homogenized using high shear homogenizer (Omni TH115), in a 1:10 (w/v) of ice-cold freshly prepared lysis buffer consisting of PBS containing 1 μg/ml Leupeptin, 1mM PMSF, and 1mM EDTA. The cortical homogenate was centrifuged at 14,000xg for 20 minutes at 4°C in a microcentrifuge. Fifty microliters of the resulting supernatant was loaded per well of the MSD plate, and IL-1β and TNFα levels determined by MSD assay. Cytokine levels in the cortex were normalized to the total amount of protein in the sample loaded as determined by BCA Protein Assay (Pierce). For serum cytokine measurements, 25 μl of undiluted serum was loaded per well of the MSD plate, and data expressed as pg/ml. The detection limits of the MSD assays are 3.4 pg/ml for TNFα and 1.5 pg/ml for IL-1β in serum/plasma.

### Statistics

Statistical analysis was conducted using GraphPad prism software version 5 (GraphPad Software, San Diego California USA, http://www.graphpad.com). Calculations of IC_50 _values were made using a nonlinear regression with a variable Hill slope, with the data normalized to the positive control to fit the top and bottom plateaus. Unless otherwise indicated, values are expressed as mean ± SEM. Groups of 2 were compared by unpaired T-test. Groups of 3 or more were compared by One-way analysis of variance (ANOVA), followed by Bonferroni Multiple Comparison Test. Significance was defined as a p < 0.05.

## Results

### The p38α MAPK inhibitor, 069A, suppresses LPS-induced cytokine up-regulation in the BV-2 microglial cell line

The ability of the small molecule p38α MAPK inhibitor, compound 069A, to inhibit proinflammatory cytokine up-regulation was tested in the mouse microglial BV-2 cell line. BV-2 cells were treated for 18 hrs with a standard activating stimulus, LPS (100 ng/ml), in the absence or presence of increasing concentrations of 069A (0.9 μM to 30 μM), and then levels of IL-1β and TNFα were measured. The results of four independent experiments are shown in Figure 1A, B. Compound 069A produced a concentration-dependent inhibition of both IL-1β (Figure 1A) and TNFα (Figure 1B) levels in LPS-treated cells, reaching greater than 80% inhibition at the 30 μM concentration. The calculated IC_50 _for suppression of IL-1β was 3.7 μM, with a 95% confidence interval of 3.1 to 4.4 μM. The calculated IC50 for suppression of TNFα was 4.5 μM, with a 95% confidence interval of 3.8 to 5.3 μM.

To provide insight into the temporal action of 069A, we asked whether the compound would be effective when given after stimulation or whether 069A needs to be added to cells before or at the same time as stimulus. We stimulated BV-2 cells with LPS (100 ng/ml), then added 069A (at the IC_50 _concentration of 4 μM) at 30min intervals up to 2 hrs after LPS treatment. IL-1β and TNFα levels were then measured at 18 hrs after LPS addition. As expected, 4 μM of 069A given at the same time as LPS (time 0) resulted in approximately 50% inhibition of IL-1β (Figure 1C) and TNFα (Figure 1D). Interestingly, there was no loss in effectiveness when 069A was given even 2 hrs after the LPS stimulation. This was confirmed in two independent experiments.

### LPS-induced phosphorylation of p38 substrates, MK2 and MSK1, is reduced selectively by treatment with 069A

To confirm that 069A was inhibiting the p38α MAPK pathway and to examine the selectivity of the downstream signaling responses, BV-2 cells were analyzed by western blot for the levels of activated (phosphorylated) p38 target proteins. Figure 2 shows results of five independent experiments. As expected, cells treated with 100 ng/ml LPS for one hr showed a significant increase (p < 0.05) in the levels of the phosphorylated (activated) forms of the four p38 substrates we examined: mitogen-activated protein kinase-activated protein kinase 2 (MK2), mitogen- and stress-activated kinase 1 (MSK1), cyclic AMP response element binding protein (CREB), and activating transcription factor-2 (ATF-2). We explored the ability of 069A to suppress the LPS-induced phosphorylation of the p38 substrates. As shown in Figure 2A, there was strong concentration-dependent inhibition (IC_50 _of 2.8 μM) of phosphorylated (p)-MK2, a kinase whose activity is dependent on activation by p38α [28]. MSK1 is a kinase that can be phosphorylated by both p38 and Erk1/2 [29]. At a concentration of 1.9 μM and higher, 069A significantly reduced the amount of p-MSK1, with an IC_50 _of 6.1 μM (Figure 2B). CREB, which is a downstream target of MSK1, was also significantly inhibited by 069A, but only at a compound concentration of 15 μM or higher (Figure 2C). ATF2 is a target of JNK [30] as well as p38α/β [31,32], and 069A did not inhibit p-ATF2 levels (Figure 2D). These data demonstrate that 069A shows selectivity in its engagement of downstream substrates in the p38 MAPK pathway.

### p38α MAPK is important for the cytokine response to different TLR ligands

To confirm that the effects of 069A were not limited to LPS stimulation, we tested the ability of 069A to suppress production of cytokines in BV-2 cells treated with a panel of TLR ligands. We chose to examine ligands for TLR2, TLR3, TLR4, TLR7/8, and TLR9 based on three criteria. First, the expression of these receptors has been shown to be elevated in areas of the brain associated with CNS neurodegenerative disease pathology [33]. Second, microglia have been shown to express these receptors [34]. Finally, ligands for these TLRs induced a reproducible cytokine response in BV-2 cells. As shown in Figure 3A, the TLR ligand panel produced a significant increase in IL-1β, with the exception of the TLR3 ligand poly(I:C). High molecular weight and low molecular weight versions of poly(I:C) were tested, but neither version resulted in a significant increase in IL-1β. All the TLR ligands tested, including the TLR3 ligand poly(I:C), produced a large and significant (p < 0.0001) TNFα cytokine response (Figure 3B). Both versions of poly(I:C) produced similar increases in TNFα; only data from the high molecular weight version of poly(I:C) is shown.

We tested whether 069A would be effective at blocking the increase in cytokines induced by TLR ligands. As shown in Figure 3C, 4 μM 069A significantly (p < 0.001) decreased the amount of IL-1β that was produced by the BV-2 cells in response to each TLR ligand tested. The percent inhibition by 069A with TLR2, TLR7/8, and TLR9 ligands was not significantly different from the inhibition seen with LPS, the TLR4 ligand. Similar results were seen with TNFα production, where 069A treatment resulted in a significant inhibition (p < 0.001) of TNFα levels in response to all the TLR ligands (Figure 3D).

### The p38α MAPK pathway is engaged in primary microglia and contributes to cytokine up-regulation

To examine the generality of the response of stressor-stimulated microglia to the p38α inhibitor 069A and confirm that the results were not specific to the BV-2 cell line, we tested primary microglia stimulated with either a TLR ligand (LPS) or a non-TLR ligand (Aβ_1-42_). The LPS-induced increases in IL-1β (Figure 4A) and TNFα (Figure 4B) levels in rat primary microglia were significantly (p < 0.001) inhibited by 069A (15 μM). Similar results were obtained with Aβ_1-42 _(Figure 4A, B), demonstrating that the p38α pathway is not limited to TLR ligand activations. We also found that phosphorylation of the p38α MAPK substrates MK2 (Figure 4C) and MSK1 (Figure 4D) was increased with ligand activation of primary microglia, and that 069A treatment significantly decreased the levels of p-MK2 and p-MSK1.

**Figure 5:**
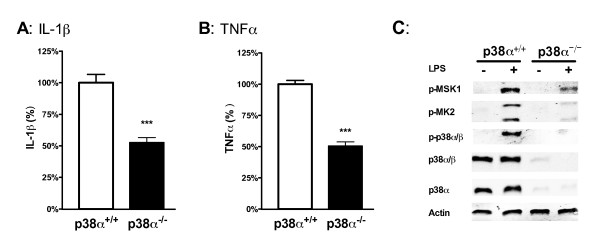
**Microglia deficient in p38α MAPK show a reduced cytokine response to LPS**. Primary microglia isolated from either p38α conditional KO mice (p38α^-/-^; black bar) or wild-type littermates (p38α^+/+^; white bar) were stimulated with 1ng/ml LPS for either 18 hrs (for cytokine measurements) or 1 hr (for western blots). There was a significant reduction in the IL-1β **(A) **and TNFα **(B) **response to LPS in microglia from the p38α KO mice compared to wild-type microglia. **(C) **There was also substantially reduced LPS-induced phosphorylation of p38α substrates MSK1 and MK2 in p38α^-^/^- ^microglia compared to p38α^+/+ ^microglia. The loss of p38α in the KO microglia was confirmed by little or no reactivity with a phospho-p38α/β antibody, a p38α/β antibody and a p38α-selective antibody. Asterisk denotes significance (*** = p < 0.001) for p38α^+/+ ^microglia (white bar) compared to p38α^-/- ^microglia (black bar). Responses of the wild-type microglia were normalized to 100%. Data represent three independent experiments.

To complement the pharmacological experiments and confirm the importance of p38α, primary microglia were isolated from p38α conditional knockout mice, where p38α is genetically deficient in macrophages/microglia. Control cells were microglia isolated from the wild-type littermates. Upon stimulation of the microglia cultures with 1ng/ml of LPS, we found an approximately 50% reduction in the levels of IL-1β (Figure 5A) and TNFα (Figure 5B) in the p38α-deficient microglia compared to the wild-type microglia. Using a p38α-selective antibody, we confirmed by Western blots that essentially no p38α was detected in the knockout microglia (Figure 5C). There also was no compensatory increase in p38β in these microglia, as antibodies that react with both p38α and p38β showed little or no detectable reactivity with the knockout microglia (Figure 5C). Finally, there were substantially reduced levels of p-MSK1 or p-MK2 in the LPS-treated knockout microglia, confirming that there is little to no activation of the p38α pathway in these microglia. Altogether, these pharmacological and genetic data demonstrate that the p38α MAPK pathway is activated in ligand-stimulated primary microglia, and that this pathway contributes to the microglial cytokine up-regulation in response to diverse stressors.

### Oral administration of 069A suppresses cytokine up-regulation in vivo in response to LPS

**Figure 6:**
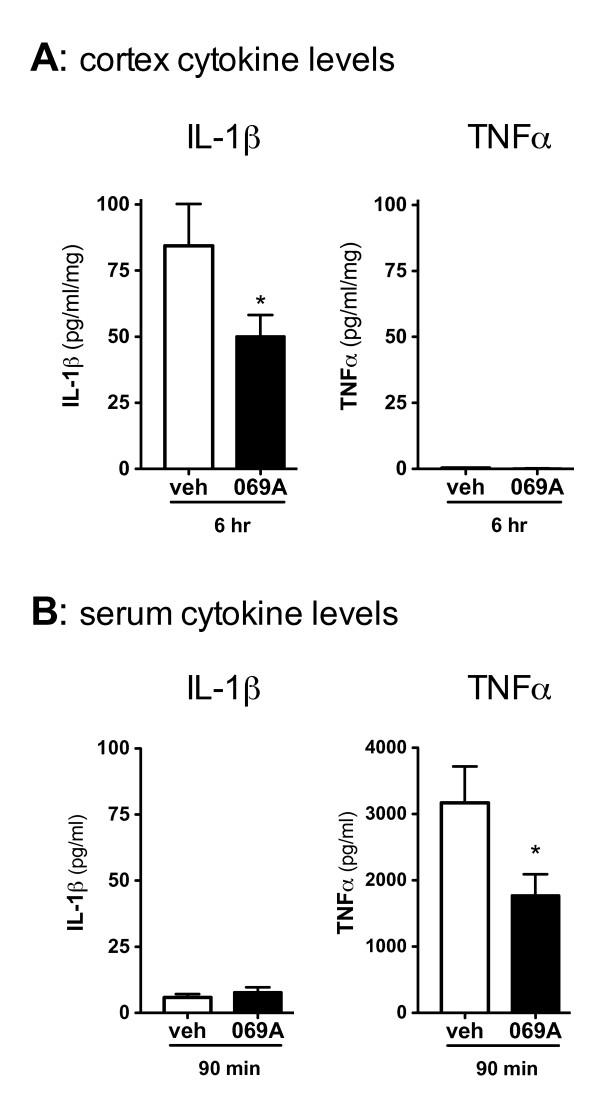
**Oral administration of 069A suppresses LPS-induced cortical IL-1β and serum TNFα *in vivo*: C57Bl/6 mice were administered either saline vehicle or 069A (5mg/kg) by oral gavage one hr prior to an intraperitoneal LPS (1mg/kg) injection**. Serum and cortex were harvested at 90 min and 6 hrs, respectively, after LPS injection. **(A) **At the 6 hr timepoint, IL-1β levels were significantly lower in the cortex of mice treated with 069A compared to vehicle-treated mice. TNFα levels in the cortex were at or near the limit of detection of the assay. **(B) **At the 90 min timepoint, TNFα levels were significantly lower in the serum of mice treated with 069A compared to vehicle-treated mice. Serum IL-1β levels were low, and there was no inhibition by 069A at this timepoint. Asterisk denotes significance (* = p < 0.05) for vehicle-treated mice (white bar) compared to 069A-treated mice (black bar). N = 5-8 per group.

LPS was used to produce an acute inflammatory response in the brain to determine if 069A given orally could reduce the CNS inflammatory response. In our mouse model, we have found that an intraperitoneal (ip) injection of LPS induces IL-1β up-regulation that peaks at 6 hrs after the LPS stimulation (data not shown). Therefore, 069A (5 mg/kg) was administered by oral gavage 1 hr before LPS stimulation, mouse brain was harvested at 6 hrs after LPS addition, and IL-1β levels in the cortex were measured. As shown in Figure 6A, 069A treatment caused a significant (p < 0.05) reduction in the amount of IL-1β produced in the cortex following LPS stimulation compared to the LPS-stimulated mice that received vehicle. IL-1β was not detectable in the non-LPS treated mice (data not shown). TNFα levels in the LPS-treated mice were near basal levels at this time point, so the ability of 069A to inhibit TNFα in cortex could not be determined.

In this acute LPS model, the effect of 069A could be to inhibit the production of IL-1β from microglia, as demonstrated in the cell culture studies. However, 069A could also be lessening the initial response in the periphery to LPS and thereby reducing the severity of the response in the CNS. To begin to address this possibility, we tested the potential of 069A to block the serum cytokine response to LPS using a 90 min time point that we have previously found to be the time of peak response of serum TNFα to an ip LPS challenge. As shown in Figure 6B, 069A did significantly reduce the serum TNFα levels in mice administered LPS for 90 min (p < 0.05). The serum IL-1β levels were low and there was no inhibition by 069A at this time point.

## Discussion

We report here several findings with important implications for the treatment of CNS inflammation. First, we show using both pharmacological and genetic approaches that the p38α MAPK isoform is sufficient for blocking a substantial portion of the IL-1β and TNFα produced by microglia following LPS stimulation. Second, using primary microglia and a microglia cell line we show that the brain-penetrant p38α inhibitor, 069A, is able to block the cytokine response to diverse, disease-relevant stimuli. Third, we demonstrate *in vivo *that a single oral dose of 069A can block the IL-1β response in the brain to a peripheral LPS insult. Fourth, we show that 069A inhibits the activation of known downstream targets of p38α, namely MK2 and MSK1, and demonstrates selectivity in the signal transduction pathways that it affects. Altogether, our data support the idea that the p38α MAPK pathway is quantitatively important for microglia proinflammatory cytokine up-regulation in response to a variety of stressors, and that the kinase may be a viable drug discovery target for CNS disorders where overproduction of proinflammatory cytokines has been implicated in disease progression.

Cytokines are inflammatory mediators that act throughout the body, including the CNS, via specific receptors and signal transduction pathways. Clinical studies and preclinical animal models have provided extensive evidence to support the hypothesis that overproduction of proinflammatory cytokines contributes to the progression of chronic neurodegenerative disorders (for review, see [11]), as well as to increased susceptibility to later-in-life disease or secondary injuries [35]. Microglia are the primary cell type in the CNS responsible for the production of cytokines. We were able to inhibit the production of two key proinflammatory cytokines, IL-1β and TNFα, with the p38α MAPK inhibitor 069A at concentrations consistent with its ability to block p38α kinase activity. Interestingly, we found that 069A was equally effective at blocking the production of cytokines when the compound was given up to two hrs after the LPS treatment; however, the mechanism underlying this observation was not pursued as part of this study. The observation that 069A also suppresses the phosphorylation of two direct p38 substrates, MK2 and MSK1, provides additional evidence of target engagement in the microglial cells. The inhibition of the active phosphorylated form of MK2 upon 069A treatment is consistent with the observation [36] that MK2 kinase activity is dependent on p38α MAPK for its phosphorylation and activation. MK2 has also been reported to directly regulate TNFα at the 3'-untranslated region (UTR) of mRNA in the AU-rich elements [37], suggesting a potential mechanistic explanation for the action of 069A in suppression of cytokine production. MSK1 can be phosphorylated and activated by both p38α MAPK and ERK1/2 [29]. However, our observation that inhibition of p38α MAPK by 069A or by genetic deletion leads to suppression of phosphorylated MSK1 in the microglia cultures suggests that the p38α MAPK pathway is playing a key role in transducing the stressor stimulus to cytokine production in microglia.

In AD-relevant models, TLR2 and TLR4 have been shown to be required for microglia cytokine response to fibrillar Aβ [38]. Microglia associated with senile plaques exhibit elevated levels of TLR2/4/5/7/9 [33]. While the role of TLRs in AD may be the best defined for any CNS disorder to date, the potentially neurotoxic role of TLRs is not limited to AD (for a recent review see: [39,40]). The inflammatory cytokine response to TLR ligands is typically through NF-κB and MAPK signal transduction pathways [41]. However, the response to the ligands can vary depending on the cell type activated. In BV-2 cells we found that 069A was highly effective at blocking the inflammatory cytokine response to TLR2/3/4/7/8/9 ligands. This suggests that p38α plays an important role in the signal transduction pathways that lead to inflammatory cytokines in microglia in response to a variety of ligands, and that this kinase may prove to be an effective convergence point that can be targeted by small molecule inhibitors, such as 069A, to block cytokine overproduction. Blocking p38α MAPK in microglia, a principle source of inflammatory cytokines such as IL-1β, may ameliorate the detrimental sequelae of increases in neuronal tau phosphorylation, synaptic loss, or other cytokine-induced neuronal damage responses [42-45]. In addition, inhibition of p38 MAPK in the neuron may also have beneficial consequences. Particularly relevant to AD, for example, are reports showing that activation of neuronal p38 MAPK contributes to Aβ-induced impairment of cortical LTP [46], and that p38 MAPK can phosphorylate tau *in vitro *at sites seen in AD brain [47,48].

It should be emphasized that microglia responses to stimuli can be neuroprotective and assist with phagocytosis or protein aggregate clearance, or can be detrimental and contribute to a progression of pathology [13,49-51]. Therefore, attempts to develop disease-modifying therapeutics that target microglial activation responses must be selective in their action (e.g., NOT be pan-suppressors of glial activation such as steroids), must consider the stage of disease progression and the relative contribution of a given endpoint or signaling pathway to the particular disease stage, and the appropriate dosing.

Dosing is the pharmacological foundation to selective therapeutic intervention. Dosing includes the amount of drug given normalized to body weight or volume and includes the therapeutic time window for administration. The desired effect of a drug, therefore, requires a combination of timing of administration based on mechanism of action and the amount administered. It is a given that all drugs will have an adverse effect at some dose, so safety with efficacy depends on finding the appropriate concentration range over which the desired effects are observed in the absence of undesired effects. However, one can also obtain pharmacological selectivity with dosing, or one pharmacological effect at one dose range and additional desired effects across a higher dose range. Examples from prior art relative to modulating inflammation are instructive in this regard. Steroids are used as anti-inflammatories, but are pleiotropic in their actions. The pleiotropic effect contributes to a diversity of pharmacological actions across efficacious doses and to a comparatively narrow therapeutic range in the absence of adverse events. In contrast, non-steroidal anti-inflammatories (NSAIDs) are more selective in their action due to their targeting of the cyclooxygenases and altering biologically active eicosanoids such as prostaglandins and thromboxanes. However, the NSAIDs require appropriate dosing for safe and effective use due to the fundamental importance of the widely distributed cyclooxgenase targets in physiological processes (e.g., [52,53]). In the case of the NSAID drug aspirin, it can be administered daily at a very low-dose for cardiovascular disease modification or 'as needed' dosing at higher concentrations for CNS symptomatic effects (for reviews, see [54,55]). It is anticipated that the pharmacodynamic (what the drug does to the body) effects of p38 MAPK inhibitors will be determined by the dosing regimen which must be empirically determined. The concentration-dependent target engagement in microglia treated with a p38 MAPK inhibitor as shown in this report is consistent with the achievement of a desired pharmacodynamic effect through appropriate dosing. However, the potential for dosing microglial product endpoints studied here must be placed in the context of other potential roles, such as effects on phagocytosis/clearance or neuronal functions, which remain to be dissected.

It is important to note that compound 069A is not a NSAID as it does not target cyclooxgenases. In terms of anti-inflammatory actions, 069A's pharmacological effects more closely resemble those of macromolecular Biological Response Modifier drugs, such as the TNFα blockers Etanercept or Infliximab, that alter inflammation-related pathology and exhibit extended pharmacodynamic effects after each administration. A goal of p38α MAPK targeted drug development for CNS indications is to generate small molecule drugs that partially mimic the biological actions of macromolecular biological response modifiers, yet are bioavailable, CNS-penetrant compounds that modulate disease-relevant endpoints. The data presented here demonstrate that pharmacological inhibition of p38α MAPK effectively suppresses the microglial cytokine upregulation response to a number of different activating ligands, raising the possibility for an extended pharmacodynamic effect due to alteration of cytokine production.

In order to explore the potential for an *in vivo *effect of 069A on cytokine levels, we screened for an effect on the proinflammatory cytokine surge induced by LPS administered *ip*. The results reveal a clear pharmacodynamic effect on brain cytokine level. Further exploration of the mechanisms for this *in vivo *effect was not done as part of this study, but deserves a cautionary comment. LPS administered *ip *will elicit a strong peripheral inflammatory response, which is then transduced to the brain via many pathways (for a recent review see: [56]). Therefore, in the LPS model, one cannot distinguish between a direct effect of a compound on microglia cytokine induction or a more indirect effect that involves reduction of the inflammatory response in the periphery which then leads to a reduction in the CNS response (or a combination of the two mechanisms). Regardless, we previously reported [18] that oral administration of 069A attenuated excessive proinflammatory cytokine production in the brain of an AD-relevant mouse model stimulated centrally with a disease-relevant stressor (oligomeric Aβ_1-42_). Therefore, viewing both our current and previous *in vivo *results in the context of the glial biology results indicates a probable direct effect on microglia as one component of the therapeutic outcome. In addition, our results provide evidence that oral administration of 069A can reduce the levels of IL-1β in the brain brought about by more than one class of *in vivo *stressor.

## Conclusions

Our results document a role for p38α MAPK as a critical regulator of IL-1β and TNFα overproduction by microglia in response to TLR ligands, and indicate that p38α MAPK also plays this key role in response to other classes of microglial activators, including Aβ_1-42_. In the context of a rapidly growing literature in CNS drug discovery, the findings presented here suggest that therapeutic strategies targeting p38α MAPK pathways can be potentially complementary to other disease progression approaches to complex diseases and susceptibility to injury where perturbation of glial-neuronal homeostasis is involved in the mechanism of pathophysiology.

## Abbreviations

(AD): Alzheimer's disease; (Aβ): β-amyloid; (CNS): central nervous system; (069A): MW01-2-069A-SRM; (IL-1β): interleukin 1β; (TNFα): tumor necrosis factor-α; (LPS): Lipopolysaccharides; (TLR): Toll like receptor; (MAPK): Mitogen-Activated Protein Kinase; (MSK1): Mitogen- and stress-activated kinase 1; (MK2): Mitogen-activated protein kinase-activated protein kinase 2; (ATF2): Activating transcription factor-2; (JNK): c-Jun NH2-terminal protein kinase; (ERK): extracellular signal-regulated kinase; (p): phosphorylated.

## Competing interests

DMW and LVE are principal investigators on project funding from the NIH and non-profit disease foundations with development of CNS new molecular entities as the long-term goal. Patents and patent applications covering novel compounds, including the one described here, have been filed by Northwestern University's technology transfer office and licensed to industry.

## Authors' contributions

ADB, DMW, LVE designed the research studies. ADB, BX, LA, ERD performed the experiments. ADB, DMW and LVE drafted the manuscript with the assistance of the other authors. All authors read and approved the final manuscript.
